# Identifying representative drug resistant mutants of HIV

**DOI:** 10.1186/1471-2105-16-S17-S1

**Published:** 2015-12-07

**Authors:** Xiaxia Yu, Irene T Weber, Robert W Harrison

**Affiliations:** 1 Department of Computer Science, Georgia State University, 34 Peachtree Street, Atlanta, GA, USA 30303; 2 Department of Biology, Georgia State University, Petit Science Center, Atlanta, GA, USA 30303

## Abstract

**Background:**

Drug resistance is one of the most important causes for failure of anti-AIDS treatment. During therapy, multiple mutations accumulate in the HIV genome, eventually rendering the drugs ineffective in blocking replication of the mutant virus. The huge number of possible mutants precludes experimental analysis to explore the molecular mechanisms of resistance and develop improved antiviral drugs.

**Results:**

In order to solve this problem, we have developed a new algorithm to reveal the most representative mutants from the whole drug resistant mutant database based on our newly proposed unified protein sequence and 3D structure encoding method. Mean shift clustering and multiple regression analysis were applied on genotype-resistance data for mutants of HIV protease and reverse transcriptase. This approach successfully chooses less than 100 mutants with the highest resistance to each drug out of about 10K in the whole database. When considering high level resistance to multiple drugs, the numbers reduce to one or two representative mutants.

**Conclusion:**

This approach for predicting the most representative mutants for each drug has major importance for experimental verification since the results provide a small number of representative sequences, which will be amenable for *in vitro *testing and characterization of the expressed mutant proteins.

## Background

AIDS (Acquired Immunodeficiency Syndrome) is one of the most severe pandemic diseases, and approximately 35.5 million people were infected in the year 2012 [1]. It has been almost three decades since the first case of AIDS was found in US and the cause of AIDS was identified as HIV (Human Immunodeficiency Virus) [2]. Currently, a total of 26 licensed drugs are used in anti-AIDS therapy [3]. These drugs target different steps during the HIV life cycle, including viral entry, reverse transcription, integration and maturation. HIV protease (PR) is the enzyme responsible for processing viral precursor proteins after budding of virus from the host cell during the maturation stage of the viral life cycle [4]. PR inhibitors block the proteolytic activity, preventing formation of the infectious virus [5,6]. HIV reverse transcriptase (RT) converts the viral RNA genome into DNA during the early stages of the HIV life cycle. The nucleoside analog zidovudine (AZT), which inhibits RT, was the first FDA approved anti-AIDS drug [7,8]. The HIV RT inhibitors can be categorized into two classes: Nucleotide analog reverse transcriptase inhibitors (NRTIs) and non-nucleoside reverse transcriptase inhibitors (NNRTIs). NRTIs are structural analogs of nucleotides, and compete with the enzyme's natural substrates during the reverse transcription step. NNRTIs specifically target a separate site on HIV-1 RT to decrease its enzymatic activities [9].

During typical anti-AIDS treatment, which is often referred to as highly active antiretroviral therapy (HAART), three or more antiretroviral drugs chosen from different categories are given to patients. Such treatment extends the lifespan of the patients [10].

However, since HIV is a member of the retrovirus family [11], its genomic information is carried by RNA [11,12]. Due to the lack of proofreading by RT [13] and the high replication rate of as many as 10^9 ^viral particles daily [14], drug resistance is one of the most severe challenges for successful long-term AIDS therapy [15,16]. Drug pressure causes the selection of resistant viral strains, which can replicate in the presence of drugs [17,18]. This drug resistance can cause the failure of antiviral therapy. Two strategies have been pursued to overcome the challenge of drug resistance. First, in the clinic, genotyping of the infecting virus is used to guide the choice of effective drugs for therapy. Drug resistance can be predicted from genotype data by a variety of algorithms [19-22], including our approach of applying a structure vector from Delaunay triangulation [23,24]. Second, research to understand the molecular mechanisms of drug resistance is important and could help in the design of new drugs for improved anti-AIDS therapy.

Several possible mechanisms have been described for drug resistance [25,26]. Laboratory studies can only be performed on a small number of mutants. However, a huge number of possible mutants can occur, since HIV has a high mutation rate of about 10^-4 ^to 10^-5 ^mutations per nucleotide and cycle of replication [26] and a naturally polymorphic genome. Taking HIV PR as an example, mutations of more than thirty different residues have been associated with PR inhibitors [16]. Moreover, multiple mutations accumulate as the virus evolves higher levels of resistance [27,28]. For instance, we have studied a PR mutant with 20 substitutions, which shows more than 1000-fold worse binding to inhibitors darunavir (DRV) and saquinavir (SQV) compared to wild-type PR [29]. Therefore, considering the huge number of possible mutants, can a tractably small number of mutants be identified as the most representative of high level resistance? Answering this question could save both time and money, and facilitate the study of drug resistant mechanisms.

One approach to selecting a small number of meaningful mutants uses the Mean shift clustering, which was first introduced in 1975 by Fukunaga and Hostetler [30] for the purpose of seeking the mode of a density function in the given sample set. Fukunaga and Hostetler [30] also suggested that mean shift clustering is an instance of gradient ascent by using decreasing distance functions, which often referred as a kernel, from a given point to a point in the sample set. This algorithm became more widely used after 1995 when Cheng [31] developed a more generalized formulation. By clarifying the relationship between mean shift and the optimization, the algorithm could potentially be applied on clustering and global optimization problems by declaring each mode as representative of one cluster and assigning each data point to the mode it converges to. Applications of the mean shift algorithm range over image/video segmentation, image representation/retrieval, discontinuity-preserving smoothing [32,33], higher level tasks like appearance-based clustering [34,35], tracking including blob tracking [36] and face tracking [37], shape detection and recognition [38]. Subsequently, applications of this algorithm were extended to other fields like biology. These applications include analysis of structural variation in genomes [39], DNA microarray analysis [40], and time-warped gene expression analysis [41].

In this paper, we have proposed a new algorithm based on the non-parametric iterative mean shift and our recently reported protein encoding method to extract the most representative drug resistant mutants from the Stanford HIV database [42].

## Results

Mean shift clustering, multiple regression and quantile analysis were performed on the data for both HIV-1 PR and RT mutants whose sequences and structures were encoded by Delaunay triangulation.

### Mean shift clustering on HIV protease inhibitor resistance

**Figure 1:**
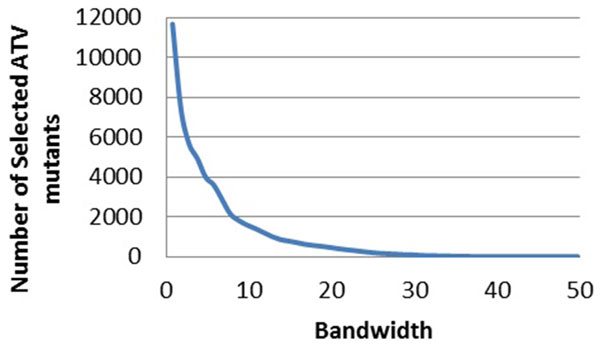
**The relationship between the bandwidths and the number of selected mutants for PIs**. The bandwidth is plotted against the number of selected mutants. The trend line is shown in blue. Plots show regression for drug resistance to ATV.

After each of the mutated sequences was represented by a 210-dimensional vector, we performed the mean shift clustering on the drug resistance data to select the most representative mutants. Data were analyzed for the PR inhibitors atazanavir (ATV), nelfinavir (NFV), ritonavir (RTV), indinavir (IDV), lopinavir (LPV), tipranvir (TPV) and saquinavir (SQV). The results show that the larger the bandwidth, the smaller number of mutants was selected. The plot for the PR inhibitor ATV is given as a representative example in Figure 1.

### Mean shift clustering on HIV reverse transcriptase inhibitor resistance

**Figure 2:**
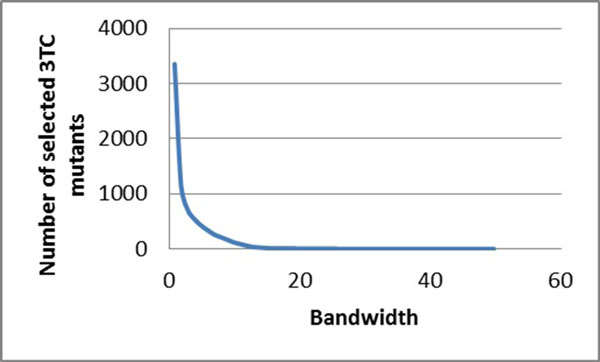
**The relationship between the bandwidths and the number of selected mutants for NRTIs**. The bandwidth is plotted against the number of selected mutants. The trend line is shown in blue. Plots show regression for drug resistance to 3TC.

**Figure 3:**
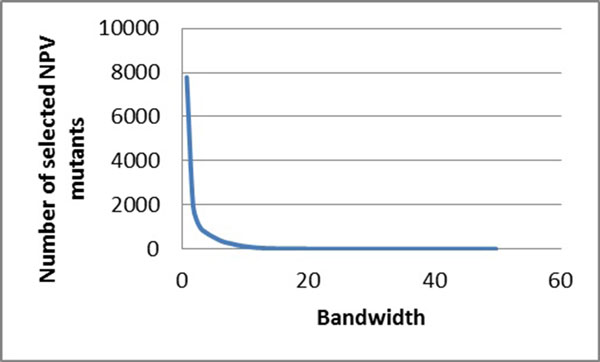
**The relationship between the bandwidths and the number of selected mutants for NNRTIs**. The bandwidth is plotted against the number of selected mutants. The trend line is shown in blue. Plots show regression for drug resistance to NPV.

Similarly, mean shift clustering was performed on the drug resistance data for HIV-1 RT inhibitors. The bandwidth and the selected numbers of mutants are compared for the RT inhibitors, including the NRTIs lamivudine (3TC) (Figure 2), abacavir (ABC), zidovudine (AZT), stavudine (D4T), didanosine (DDI) and tenofovir (TDF) (Figure 2), and the NNRTIs nevirapine (NPV) (Figure 3), delaviridine (DLV), and efavirenz (EFV).

### Multiple regression on HIV protease inhibitor resistance

**Figure 4:**
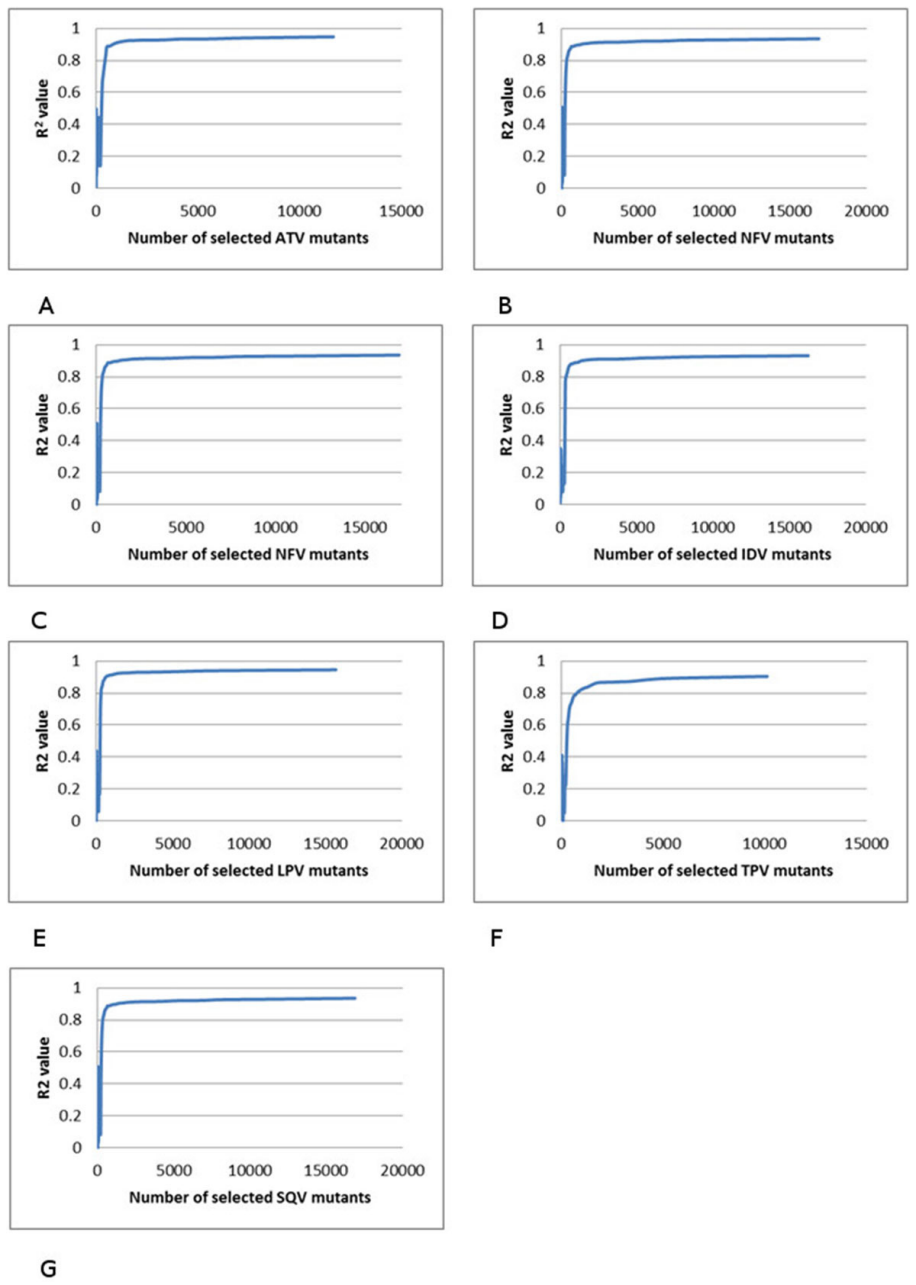
**The relationship between the multiple regression results and the number of selected mutants**. The R2 value is plotted against the number of selected mutants. The trend line is shown in blue. Plots show regression for resistance to drugs: (A) ATV, (B) NFV, (C) RTV, (D) IDV, (E) LPV, (F) TPV, and (G) SQV.

Afterwards, a multiple regression was applied to the selected mutants to evaluate the selected results. The R^2 ^values for relative resistance were plotted against the number of selected mutants as shown in (Figure 4) for the PR inhibitors ATV, NFV, RTV, IDV, LPV, TPV and SQV. The x-axis is the number of selected mutants, while the y-axis is the R^2 ^value after applying multiple linear regression on selected protein sequences with their drug resistant values.

### Multiple regression on HIV reverse transcriptase inhibitor resistance

**Figure 5:**
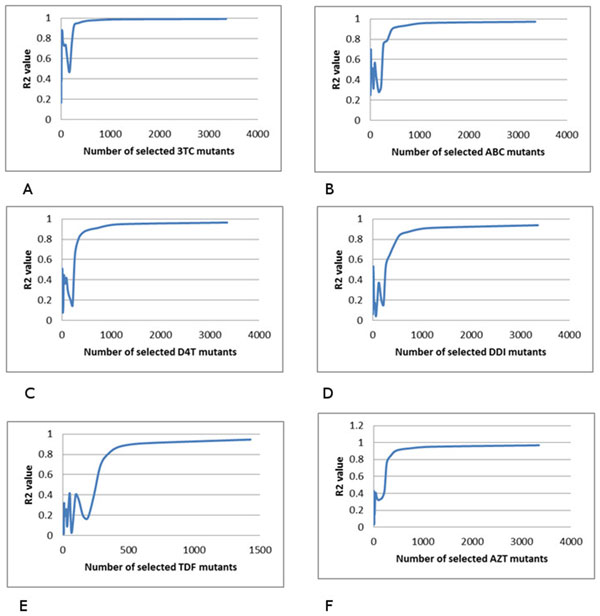
**The relationship between the multiple regression results and the number of selected mutants**. The R2 value is plotted against the number of selected mutants. The trend line is shown in blue. Plots show regression for resistance to drugs: (A) 3TC, (B) ABC, (C) D4T, (D) DDI, (E) TDF and (F) AZT.

**Figure 6:**
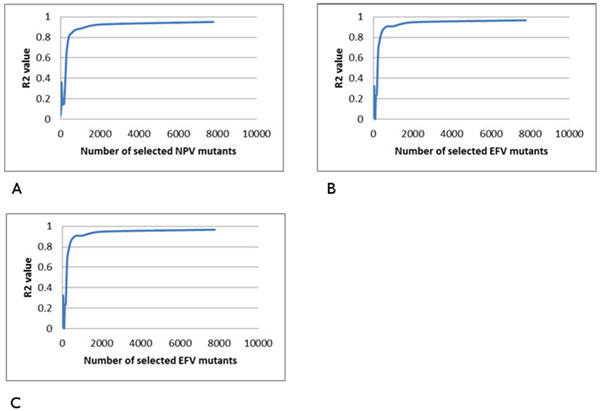
**The relationship between the multiple regression results and the number of selected mutants**. The R2 is plotted against the number of selected mutants. The trend line is shown in blue. Plots show regression for resistance to drugs: (A) NPV, (B) DLV and (C) EFV.

Multiple regression analysis was performed similarly on genotype-phenotype data for drugs inhibiting HIV-1 RT. The R^2 ^values for relative resistance were plotted against the number of selected mutants as shown in for the RT inhibitors including NRTIs 3TC, ABC, D4T, DDI, TDF and AZT (Figure 5), and NPV, DLV and EFV for NNRTIs (Figure 6).

### Bandwidth selection and multiple regression on HIV-1 PR and RT inhibitor resistance

The following experiments were performed to test the accuracy of the selected mutants with different R^2 ^results. According to the results of the above experiments, it could be inferred that the larger the bandwidth is, the fewer representative mutants are selected, and therefore the R^2 ^would be lowered. Moreover, with different R^2 ^values, the selected mutants of lower R^2 ^need to be a subset or have a large intersection with the selected mutants of the higher R^2^. Based on the above results, in this experiment, the higher R^2 ^was set to be above 0.80 while the lower R^2 ^was set to be 0.60. With this goal, the overlap group was then calculated.

The overlap group of mutants is a significant fraction of those selected for the lower R^2^, which suggests the procedure selects meaningful representative mutants correctly. The fractional overlap ranges from 0.79 to 0.94 for HIV PR inhibitors, and 0.89-0.94 for NNRIs (Tables 1, 2). For NRTIs, the results in Figure 5 show that when the number of resistant mutants increases, the R^2 ^value does not increase smoothly. There are many ripples in the plots, making it difficult to select the R^2 ^cutoff in this experiment. Therefore, analysis of NRTIs was not possible.

**Table 1:** The number of selected mutants and R^2 ^for HIV-1 PR Inhibitors.

	Selected for higher R^2^	Higher R^2^	Selected for lower R^2^	Lower R^2^	Overlap	Overlap Ratio (%)
ATV	412	0.8089	289	0.6004	260	89.97

NFV	353	0.8085	253	0.6246	235	92.89

RTV	281	0.8027	243	0.5915	228	93.83

IDV	420	0.8018	252	0.5749	217	86.11

LPV	307	0.8006	258	0.6073	242	93.80

TPV	826	0.8080	288	0.5837	227	78.82

SQV	453	0.8151	243	0.6188	215	88.48

**Table 2:** The number of selected mutants and R^2 ^for HIV-1 RT NNRTIs.

	Selected for higher R^2^	Higher R^2^	Selected for lower R^2^	Lower R^2^	Overlap	Overlap Ratio (%)
NPV	429	0.8067	273	0.6050	242	88.64

DLV	337	0.8162	257	0.6047	242	95.16

EFV	337	0.8073	243	0.6449	222	91.36

### Quantile information analysis on HIV-1 protease inhibitor resistance

In order to further analyze the mutants selected by mean shift, quantile information analysis was performed and the result indicates that the proposed algorithm could successfully cluster the datasets, and pick the potentially most drug resistant mutants from the cluster centers (Tables 3, 4). In the tables, the numbers are given for selected/total number in each bin, and R^2 ^used here is around 0.70.

**Table 3:** Number of selected mutants in each bin for PIs.

	ATV	NFV	RTV	IDV	LPV	TPV	SQV
I	189/9454	183/13711	151/12220	246/14885	152/11630	366/9921	223/14746

II	36/1179	55/2126	34/1589	35/1101	62/2087	16/87	19/910

III	18/844	22/540	11/918	10/511	31/1393	0/0	0/107

IV	9/200	7/357	6/300	14/216	13/200	0/0	3/28

V	10/39	4/21	7/304	4/14	8/333	1/1	2/94

VI	1/3	1/256	0/0	0/0	1/26	0/0	2/132

VII	3/34	0/2	2/22	1/8	3/153	0/0	0/0

VIII	1/129	0/0	0/0	1/12	2/3	0/0	1/1

IX	0/0	1/9	0/0	0/0	0/0	0/0	0/0

X	24/202	15/523	59/1299	10/99	12/444	29/219	28/1100

**Table 4:** Selected ratios in each bin for PIs (%).

	ATV	NFV	RTV	IDV	LPV	TPV	SQV
I	2.00	1.33	1.24	1.65	1.31	3.69	1.51

II	3.05	2.59	2.14	3.18	2.97	18.4	2.09

III	2.13	4.07	1.20	1.96	2.23	N/A	0.00

IV	4.50	1.96	2.00	6.48	6.50	N/A	10.7

V	25.6	19.1	2.30	28.6	2.40	100	2.13

VI	33.3	0.391	N/A	N/A	3.85	N/A	1.52

VII	8.82	0.00	9.09	12.5	1.96	N/A	N/A

VIII	0.775	N/A	N/A	8.33	66.7	N/A	100

IX	N/A	11.1	N/A	N/A	N/A	N/A	N/A

X	11.9	2.87	4.54	10.1	2.70	13.2	2.55

Bin I includes the mutants with least resistance to each inhibitors, while Bin × has the mutants with the highest resistance to the inhibitors. As shown in Table 4, the selected ratio in bin × is larger than that of bin I. This result suggests that the mutants vary more in the drug resistant category than in the non-drug resistant one.

### Quantile information analysis on HIV-1 reverse transcriptase inhibitor resistance (NRTIs)

In order to further analyse the mutants selected by mean shift, all the drug resistant mutants were grouped and separated into 10 bins based on their drug resistance value. Both the total number of mutants and the selected number of mutants were counted and recorded in each corresponding table (Tables 5, 6). In the tables, the numbers are selected/total number in each bin, and R^2 ^used here is around 0.70. Similar to PIs results, as shown in Table 6, the selected ration in bin × is larger than that of bin I.

**Table 5:** Number of selected mutants in each bin for NRTIs.

	3TC	ABC	D4T	DDI	TDF	AZT
I	11/2711	241/4780	188/3791	314/4603	265/2001	142/4079

II	0/14	13/65	51/948	17/194	1/1	19/94

III	0/1	0/0	10/23	5/25	0/0	13/253

IV	0/1	0/0	7/14	1/4	0/0	4/27

V	0/73	0/0	4/37	2/7	0/0	3/7

VI	0/57	0/0	2/17	2/9	0/0	5/30

VII	1/54	0/0	1/1	0/2	0/0	5/19

VIII	0/45	0/0	2/4	0/1	0/0	3/164

IX	0/88	0/0	1/8	1/3	0/0	0/6

X	14/1806	1/1	0/2	1/1	1/2	35/168

**Table 6:** Selected ratios in each bin for NRTIs (%).

	3TC	ABC	D4T	DDI	TDF	AZT
I	0.406	5.04	4.50	6.82	13.24	3.48

II	0.00	20.0	5.38	8.76	100	20.2

III	0.00	N/A	43.5	20.0	N/A	5.14

IV	0.00	N/A	50.0	25.0	N/A	14.8

V	0.00	N/A	10.8	28.6	N/A	42.9

VI	0.00	N/A	11.77	22.2	N/A	16.7

VII	1.85	N/A	100	0.00	N/A	26.32

VIII	0.00	N/A	50.0	0.00	N/A	1.83

IX	0.00	N/A	12.5	33.3	N/A	0.00

X	0.775	100	0.00	100	50.0	20.8

### Quantile information analysis on HIV-1 reverse transcriptase inhibitor resistance (NNRTIs)

In order to further analyze the mutants selected by mean shift, all the drug resistant mutants were grouped and separated into × bins based on their drug resistance value. Both the total number of mutants and the selected number of mutants are counted and recorded in each corresponding table. In the tables, the numbers are selected/total number in each bin, and R^2 ^used here is around 0.70. The Tables 7, 8 show the total number of mutants in the bin before and after selection.

**Table 7:** Number of selected mutants in each bin for NNRTIs.

Bin	NPV	DLV	EFV
I	157/9898	198/9476	172/9907

II	17/157	24/241	14/116

III	9/114	12/587	10/166

IV	7/56	7/35	1/24

V	9/94	4/155	2/42

VI	7/169	3/20	2/132

VII	1/1	0/0	4/26

VIII	30/293	3/73	6/48

IX	0/1	3/9	1/2

X	66/584	43/703	32/891

**Table 8:** Selected ratios in each bin for NNRTIs (%).

	NPV	DLV	EFV
I	1.59	2.09	1.74

II	10.8	10.0	12.0

III	7.90	2.04	6.02

IV	12.5	20.0	4.17

V	9.57	2.58	4.76

VI	4.14	15.0	1.52

VII	100	N/A	15.4

VIII	10.2	4.11	12.5

IX	0.00	33.3	50.0

X	11.3	6.12	3.59

### Analysis of multi-drug resistance information for the most highly resistant mutants of HIV-1 PR to NRTIs

**Figure 7:**
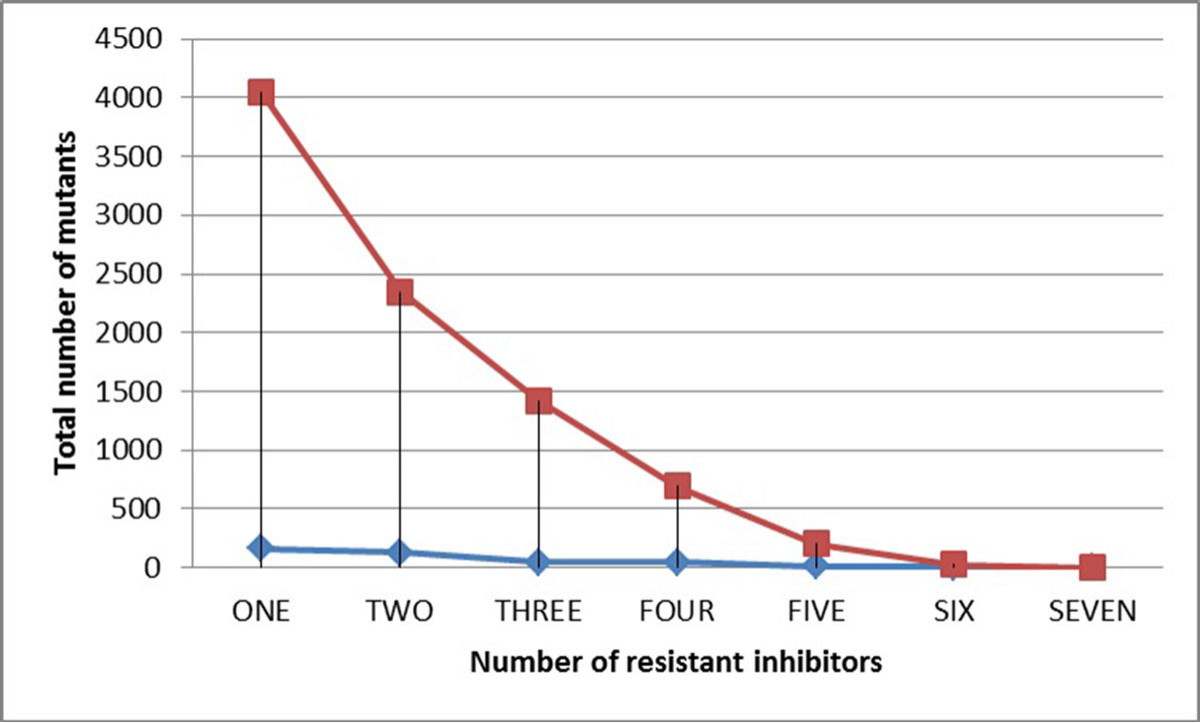
**The relation between total number of mutants and number of resistant inhibitors for PIs**. The red line shows the result for the original data in bin X; while the blue line shows the result for the selected mutants in bin X.

In order to further analyze the mutants selected by mean shift in the most drug resistant category (bin X), those mutants having resistance to multiple drugs were picked and compared. The results show that the more inhibitors a mutant is resistant to, the fewer representative mutants appear (Figure 7). The number of mutants representing high resistance to three or more PIs falls to low values of one to three, which becomes eminently verifiable by *in vitro *experiments.

### Analysis of multi-drug resistant information for the most highly resistant mutants of HIV-1 reverse transcriptase

**Figure 8:**
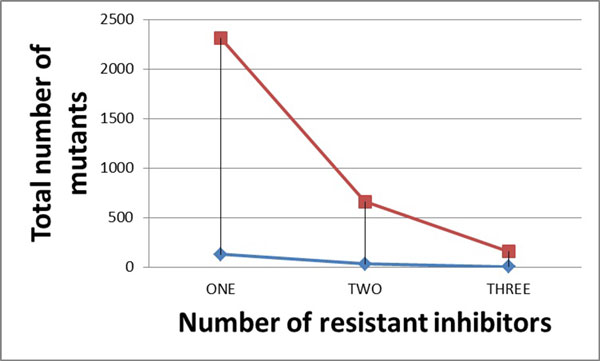
**The relation between total number of mutants and number of resistant inhibitors for NNRTIs**. The red line shows the result for the original data in bin X; while the blue line shows the result for selected mutants in bin X.

Similar results are also obtained for NNRTIs. As shown in Figure 8, the number of mutants representing high resistance to two NNRTIs falls to values of 9-12, and when all three drugs are considered, only four mutants are representative of multidrug resistance. These low numbers of mutants can be verified by *in vitro *experiments.

## Discussion

The serious problem of drug resistance arising during therapy of HIV-infected individuals can cause failure of the treatment. Many scientists are working on revealing the drug resistance mechanisms using a variety of experimental techniques. However, since there are an extremely large number of mutants, it is difficult to choose representative mutants for detailed research in the laboratory.

In this experiment, we have developed new selection algorithm based on a simple graph representation of protein structure to solve this problem. The protein structure is 3-D and can be efficiently represented by Delaunay triangulation [44]. Based on this encoding method, a mean shift was applied to select the most representative mutants. Multiple linear regression was performed to evaluate the selection results.

This selection algorithm works well on selecting drug resistant mutants from both HIV PR and RT inhibitor genotype/phenotype data. Among all the mutants, around 250 most representative mutants were selected with numbers in the range of 215 to 360 [PIs+NNRTIs] mutants selected for the different drugs. Such selection was based on the kernel bandwidth, and the goal R^2 ^value. In this experiment, the R^2 ^value was set to be above 0.60 to be considered as a successful selection. During the experiments, after selection, the multiple linear regression was applied on these selected mutants' drug resistance values, and the R^2 ^values fall in the range of 0.65 to 0.83, indicating excellent correlation. This high correlation suggests that the selected number of mutants can be further decreased if a lower target R^2 ^value was applied.

Identifying a small number of representative mutants will enable laboratory studies of the molecular mechanisms of resistance, which is currently impossible due to the huge number of possible mutants.

## Materials and methods

### Datasets and data preparation

All the genotype-phenotype datasets were downloaded from the Stanford HIV drug resistance database [42](http://hivdb.stanford.edu/pages/genopheno.dataset.html). The proposed algorithm was tested on both HIV-1 PR and HIV-1 RT resistance data sets. For HIV-1 PR, the inhibitors atazanavir (ATV), nelfinavir (NFV), ritonavir (RTV), indinavir (IDV), lopinavir (LPV), tipranvir (TPV) and saquinavir (SQV) were tested. While for HIV RT, NNRTIs nelfinavir (NPV), delaviridine (DLV), efavirenz (EFV), and NRTIs lamivudine (3TC), abacavir (ABC), zidovudine (AZT), stavudine (D4T), didanosine (DDI) and tenofovir (TDF) were tested.

All the datasets were pre-processed using the methods and the cutoff values described previously in[24]. The results of the expansion for each of the HIV-1 PR inhibitors were: a total of 16846 sequences were obtained from 1622 isolates with assays for IDV resistance; a total of 16269 sequences from 1322 isolates for LPV; a total of 10228 sequences from 744 isolates for TPV; a total of 17118 sequences from 1640 isolates for SQV; a total of 12084 sequences from 1012 isolates for ATV; a total of 17545 sequences from 1674 isolates for NFV; and a total of 16652 sequences from 1589 isolates for RTV.

For each of the HIV-1 RT inhibitors the expansion resulted in: a total of 11367 sequences were obtained from 746 isolates with assays for NPV resistance; a total of 11299 sequences from 732 isolates for DLV; a total of 11354 sequences from 734 isolates for EFV; a total of 4850 sequences from 633 isolates for 3TC; a total of 4846 sequences from 628 isolates for ABC; a total of 4847 sequences from 630 isolates for AZT; a total of 4845 sequences from 630 isolates for D4T; a total of 4849 sequences from 632 isolates for DDI; and a total of 2004 sequences from 353 isolates for inhibitor TDF.

### Encoding structure and sequence with Delaunay triangulation

The sequence and structure of the protein were represented using a graph-based encoding as described in [43]. Delaunay triangulation was used to define a graph which spanned the protein structure and defined structurally adjacent pairs of amino acid residues. Adjacent pairs of amino acids were summarized into a vector of the 210 unique pairs of the 20 standard amino acids by calculating the distance for each adjacent pair in the structure and tabulating by the types of amino acids in that adjacent pair. Only the sequences of the mutated proteins are needed and only one protein structure is necessary. As a result, all mutants are represented as vectors of the same dimensionality, which is a desired property for most of the pattern recognition algorithms. The X-ray crystal structures 3OXC for HIV-1 PR, and 2WOM for HIV-1 RT (from http://www.pdb.org) were used as templates for Delaunay triangulation.

### Regression analysis for drug resistance prediction

The genotype-phenotype datasets provide an experimentally measured drug resistance value, with respect to a certain type of drug, with each genotype. The mutations relative to a standard sequence are denoted as $x_{{}^{\text{1}}},x_{\text{2}},...x_{N};x_{i}\in {ℜ}^{\text{210}}$ where *N *is the total number of mutations and R^210 ^is the structure vector. Also the corresponding drug resistance values are denoted as the real numbers $y_{{}^{\text{1}}},y_{{}^{\text{2}}},...,y_{N};y\in {ℜ}^{}$ including 0 for the resistance value of the wild type virus. We then seek a linear model between the *x_i _*'s and *y_i_*'s by minimizing the cost function *E*:

(1) $$E\text{:}=\overset{N}{\underset{i=\text{1}}{\sum }}{\left(y_{i}-A\cdot x_{i}-b\right)}^{\text{2}}$$

with respect to the 210 dimensional vector A and scalar b.

Furthermore, in order to better utilize the available data set, we performed a *k*-fold cross-validation (in this work, k = 5). Specifically, the training set of size *N *is randomly divided into *k *groups. Among them, *k*-1 groups are utilized for constructing the linear model as in Equation (1). Then, the linear model is used to predict the drug resistance for the remaining group with *N*/*k*mutations. The predicted resistances are compared with the measured ones and the R^2 ^values are recorded. Finally, the average and standard deviation of the *k *R^2 ^values are computed.

### Mean shift clustering and bandwidth selection

The mathematical deviation of mean shift algorithm was first introduced by Fukunaga and Hostetler [30], then adapted by Cheng [31], and later extended by Comaniciu, Meer, and Ramesh [45]. The procedure of the mean shift is that, for each data point in the feature space, a gradient ascent procedure is performed until convergence. The stop points of the procedure are the local maxima of the kernal density function, which could also be considered as the center of the clustering.

Given *N *data points$\left\{x_{1},x_{2},...,x_{N}\right\}\in {ℜ}^{210}$, which could be considered as the kernel density function with Gaussian kernel $K\left(t\right)=e^{-t/2}$ for *t *≥ 0:

$$p\left(x\right)=\overset{N}{\underset{i=1}{\sum }}\pi_{i}\frac{1}{Z_{i}}K\left(d\left(x,x_{i};\sum_{i}\right)\right),$$

Where $\pi_{i}\in \left(0,1\right)$ is the mixing proportion of point *i *(satisfying$\sum_{i=1}^{N}\pi_{i}=1$), $\sum_{i}$is its covariance matrix (positive definite), $Z_{i}={\left|2\pi \sum_{i}\right|}^{1/2}$ is a normalization constant and $d\left(x,x_{i};\sum_{i}\right)={\left(x-x_{i}\right)}^{T}\sum_{i}^{-1}\left(x-x_{i}\right)$ is the Mahalanobis distance.

Among all the data points, the dense regions of these could be treated as the local maxima of *p*(*x*) and could be found by seeking stationary points$\frac{\partial p\left(x\right)}{\partial x}=0$. The mean-shift update is applied with the rule:

$$p\left(n|x\right)=\frac{\text{exp}\left(-\frac{1}{2}||\left(x-x_{n}\right)/\sigma |{|}^{2}\right)}{\sum_{n^{\prime }=1}^{N}\text{exp}\left(-\frac{1}{2}||\left(x-x_{n^{\prime }}\right)/\sigma |{|}^{2}\right)},x\leftarrow f\left(x\right)=\overset{N}{\underset{n=1}{\sum }}p\left(n|x\right)x_{n}$$

This rule corresponds to a fixed point iteration to find the expected value for the centre of a Gaussian kernel, and is computationally more efficient than a gradient based numerical optimization for this problem. The rule maps any point $x\in {ℜ}^{210}$ to a weighted mean of the points in the dataset denoted as *f*(*x*). The difference *f*(*x*)-*x *is the mean shift vector and is clearly of zero magnitude at convergence.

The mean shift algorithm is non-parametric and the resolution of the clustering is determined by the kernel bandwidth σ. The initial step is to find the range of the bandwidth. Following that, by choosing different bandwidths, different numbers of mutants were selected. A multiple regression was performed to evaluate the selected results.

### Quantile information analysis

All the drug resistant mutants were grouped and separated into 10 bins based on their drug resistance value. For example, about ATV, their resistance values range from 0 to 700. Therefore, those mutants with resistance value between 0 and 70 were put into bin I, those with resistance value between above 70 and below 140 were put into bin II, and so on.

After splitting all the data into ten bins, both the total number of mutants and the selected number of mutants were counted and recorded in each corresponding table. For each bin, the number of mutants before and after the selection was calculated and compared. Moreover, the selected ratio is also calculated.

### k-fold validation

In order to fully use all the data, a k-fold cross-validation was performed in all the experiments for all the drugs. Specifically, we randomly choose (*k*-1)/*k *of all the sequences (some are drug resistant, while others are non-drug resistant) for training the classifier and the remaining 1/*k *data are used for testing. These tests used *k *= 5. Independent randomly selected k-folds were chosen throughout the study to avoid bias in the results. The apparent polymorphism in the original sequence data requires extra care when generating k-fold data sets for testing or training. When a sequence was removed from a k-fold in generating a testing or training dataset, all derived instances of that sequence were removed as well. This ensures that the individual k-fold datasets are truly independent from each other and thus ensures that the estimated accuracies are meaningful. The R^2 ^values were averaged over the k-folds.

## Competing interests

Authors declare that they have no competing interests.

## Authors' contributions

All authors designed the experiments. XY and RWH designed the algorithms. XY implemented the algorithms and ran the predictions. All authors interpreted the results and wrote the manuscript. All authors read and approved the final manuscript.

## Declarations

Publication of this article was funded by the National Institutes of Health grant GM062920 (ITW, RWH).

This article has been published as part of *BMC Bioinformatics *Volume 16 Supplement 17, 2015: Selected articles from the Fourth IEEE International Conference on Computational Advances in Bio and medical Sciences (ICCABS 2014): Bioinformatics. The full contents of the supplement are available online at http://www.biomedcentral.com/bmcbioinformatics/supplements/16/S17.
